# Neurologic Music Therapy Training for Mobility and Stability Rehabilitation with Parkinson’s Disease – A Pilot Study

**DOI:** 10.3389/fnhum.2015.00710

**Published:** 2016-01-26

**Authors:** Anna A. Bukowska, Piotr Krężałek, Elżbieta Mirek, Przemysław Bujas, Anna Marchewka

**Affiliations:** ^1^Department of Occupational Therapy, The University of Physical Education in KrakowKrakow, Poland; ^2^Department of Clinical Rehabilitation and Laboratory of Pathology of the Musculoskeletal System, The University of Physical Education in KrakowKrakow, Poland; ^3^Department of Physiotherapy, The University of Physical Education in KrakowKrakow, Poland; ^4^Section of Rehabilitation in Neurology and Psychiatry, The University of Physical Education in KrakowKrakow, Poland; ^5^Department of Theory of Sport and Kinesiology, The University of Physical Education in KrakowKrakow, Poland

**Keywords:** Parkinson’s disease, gait, stability, neurologic music therapy

## Abstract

Idiopathic Parkinson’s Disease (PD) is a progressive condition with gait disturbance and balance disorder as the main symptoms. Previous research studies focused on the application of Rhythmic Auditory Stimulation (RAS) in PD gait rehabilitation. The key hypothesis of this pilot study, however, assumes the major role of the combination of all three Neurologic Music Therapy (NMT) sensorimotor techniques in improving spatio-temporal gait parameters, and postural stability in the course of PD. The 55 PD-diagnosed subjects invited to the study were divided into two groups: 30 in the experimental and 25 in the control group. Inclusion criteria included Hoehn and Yahr stages 2 or 3, the ability to walk independently without any aid and stable pharmacological treatment for the duration of the experiment. In order to evaluate the efficacy of the chosen therapy procedure the following measures were applied: Optoelectrical 3D Movement Analysis, System BTS Smart for gait, and Computerized Dynamic Posturography CQ Stab for stability and balance. All measures were conducted both before and after the therapy cycle. The subjects from the experimental group attended music therapy sessions four times a week for 4 weeks. Therapeutic Instrumental Music Performance (TIMP), Pattern Sensory Enhancement (PSE) and RAS were used in every 45-min session for practicing daily life activities, balance, pre-gait, and gait pattern. Percussion instruments, the metronome and rhythmic music were the basis for each session. The subjects from the control group were asked to stay active and perform daily life activities between the measures. The research showed that the combination of the three NMT sensorimotor techniques can be used to improve gait and other rhythmical activities in PD rehabilitation. The results demonstrated significant improvement in the majority of the spatiotemporal gait parameters in the experimental group in comparison to the control group. In the stability tests with eyes closed, substantial differences were revealed, indicating improvement of proprioception (the sense of body position and movement). These findings suggest a new compensatory strategy for movement and postural control through the use of the auditory system.

## Introduction

Idiopathic Parkinson’s disease (PD) is one of the most common, progressive, degenerative conditions of the central nervous system. It causes significant physical disability and leads to impairment in cognitive functions. PD affects 1% of people over the age of 60 in all developed countries. About 90,000 cases are currently registered in Poland (De Rijk et al., 1995; Friedman, 2005; Drapała et al., 2014; Opara, 2014).

The impairment of automatic and rhythmic movements is described in the pathophysiology associated with PD. The disease is caused by disappearance of dopamine production and leads to changes in the central nervous system, impairing the neural networks including the basal ganglia and supplementary motor areas. The consequences of progressing PD are increasingly felt by patients as an abnormal gait pattern, balance problems and difficulties in performing daily life activities. It leads to alterations in functioning, which results in a lowering of the quality of life (Freeman et al., 1993; Bergman and Deuschl, 2002; Martin et al., 2002; Purves et al., 2004). Typical changes in walking patterns among the PD population are described as gait slowness with the reduction or absence of reciprocal arm swing and increased double support. Furthermore, motion amplitude scaling is impaired resulting in the inability to generate sufficient step length. Gait changes in PD also include asymmetry between the sides of the body and a reduction of bilateral coordination. Deprivation of ability to produce internal walking rhythm causes an increase of asymmetry between steps (Blin et al., 1990; Hausdorff, 2009; Peterson et al., 2012). According to the Hoehn and Yahr (1967) scale, slight changes in balance are noticeable from the very beginning of the disease progression. However, considerable postural instability, based on proproception, is described in the progression of the disease between second and third H&Y stage. It engenders balance impairment, not only during gait but also in standing position (Hoehn and Yahr, 1967; Konczak et al., 2009).

Despite the degenerative nature of PD, pharmacological treatment and proper rehabilitation enable a patient’s well-functioning and active participation in daily life for years after the diagnosis. Research and clinical experience indicate the significance of the appropriate selection of therapeutic interventions, exercises, and stimulation for an optimal effect which can be translated into a better quality of life for PD patients (Keus et al., 2004, 2007; Aragon and Kings, 2010). The current data shows that approximately 1% of people of 60 years of age in Poland are annually diagnosed with PD. The aging population may lead to a significant increase in numbers of patients with this disease in the future (Drapała et al., 2014). Therefore, it is crucial to search for new therapeutic solutions.

The use of music in neurorehabilitation is grounded in neurophysiological theories, and research on the influence of music on cognitive processes and motor learning principles (Bayona et al., 2005; Kitago and Krakauer, 2012). The therapeutic approach established 20 years ago in the US called Neurologic Music Therapy (NMT) is known as an effective approach in neurorehabilitation (Thaut, 2005). NMT concepts distinguish three sensorimotor techniques, with motor skills improvement as an overall goal. The first one, Rhythmic Auditory Stimulation (RAS), is a technique that aims to develop and maintain a physiological rhythmic motor activity (gait) through rhythmic auditory cues. This technique has been proven effective for gait rehabilitation in PD (McIntosh et al., 1997; Thaut et al., 1998; Thaut and Rice, 2014). The second technique is Patterned Sensory Enhancement (PSE). The objective of this technique is to facilitate movements associated with the activities of daily life, not necessarily rhythmical in nature. PSE uses complex music elements: pitch, dynamics, harmony, meter, and rhythm to enhance and organize movement patterns in time and space, and to favorably affect the activity, muscle coordination, strength, balance, postural control and range of motion (Thaut, 2014a). The last technique, Therapeutic Instrumental Music Performance (TIMP), employs musical instruments as a task orientation training to simulate and facititate functional movements. The technique most commonly uses percussion instruments, playing them in a traditional or non-traditional way to improve range of motion, limb coordination, postural control, dexterity, body perception, and sensation (Thaut, 1999; Mertel, 2014). In order to optimize the music therapy process, NMT uses the Transformational Design Model (TDM) to translate theoretical knowledge into clinical practice. It promotes effective assessment, design and implementation of therapeutic music interventions (Thaut, 2005, 2014b).

The purpose of this pilot study was to evaluate the efficacy of music and rhythm for mobility and balance in a group of patients with PD. The key hypothesis assumed the major role of the combination of all three NMT sensorimotor techniques in improving spatio-temporal gait parameters and postural stability in the course of PD. The research question aimed to find an answer to how effective the combination of TIMP, PSE, and RAS are in the motor treatment of PD patients.

## Materials and Methods

### Participants

Fifty five subjects diagnosed with PD participated in the research project. All of them were recruited from the database of the Neurology and Neurosurgery Clinic of Jagiellonian University in Krakow. The inclusion criteria included: informed consent for participation in the experiment, Idiopathic PD diagnosed by a neurologist, stages two and three of the disease according to Hoehn and Yahr (1967; H&Y), the ability to walk independently without any aid for at least 6 m × 8 m distance, and stable pharmacological treatment for the duration of the experiment. The exclusion criteria were: lack of informed consent for participation in the experiment, musculoskeletal injuries (e.g., fractures and prosthesis), diagnosis of dementia including Alzheimer’s disease (*MMSE* < 25), and frequent changes in medications. The subjects displayed similar symptoms due to the duration and severity of the condition and were randomly assigned into two groups. The experimental group E (*n* = 30 individuals) were involved in the NMT program. The average age in this group of patients was 63.4 years. In the control group K (*n* = 25 individuals) participants were asked to maintain their daily life activities (changing of position, walking, walking stairs). The average age of the control group was 63.44 years. The characteristics in each group are presented in **Table 1.**

**Table 1:** The charakteristic of groups: the experimental and the control.

Characteristic		Experimental group		Control group		*p*
				
		*n* mean (*SD*)	% median (min-max)	*n* mean (*SD*)	% median (min-max)	
Gender	Female	15	50,00	10	40,00	*p* = 0,639
	Male	15	50,00	15	60,00	
Age [years]		63,4 (10,61)	64 (28–80)	63,44 (9,67)	63 (46–88)	*p* = 0,813
Duration of disease [years]		5,5 (3,9)	4 (1–14)	6,76 (4,32)	5 (1–18)	*p* = 0,151
H&Y scale	2	17	56,67	14	56,00	*p* = 1
	3	13	43,33	11	44,00	
Weaker body side	Left	20	66,67	17	68,00	*p* = 1
	Right	10	33,33	8	32,00	


The NMT sessions were held in the Department of Rehabilitation at the Neurology and Neurosurgery Clinic of Jagiellonian University in Krakow. The research was registered in The Ministry of Science and Higher Education, Poland. The clinical trial was approved by the Bioethical Committe of The Supreme Medical Council in Krakow, Poland.

### Methods

In order to evaluate the efficacy of the chosen therapy procedure the following measures were applied: Optoelectrical 3D Movement Analysis System BTS Smart to obtaine gait parameters and Computerized Dynamic Posturography CQ Stab to measure stability. All measures were conducted directly before and directly after 4 weeks for the experimental group (therapy cycle) and for the control group (Motion Analysis Lab, Department of Physiotherapy, University of Physical Education in Krakow, Poland).

### Gait Assessment

To measure the temporal and spatial gait parameters (stance and swing phase, double support, stride time and cadence, step and stride length, velocity and step width) Optoelectrical 3D Movement Analysis System BTS Smart was used (Davis et al., 1991; Marchewka, 2005; Rutowicz et al., 2005; Cygoń, 2011). BTS SMART allows to capture the movement with a frequency of 70 Hz. The system consists of six analog cameras with infrared light and BTS SMART-Analyzer software. Before the survey anthropometric measures, such as body height and weight, width of pelvis, depth of pelvis, diameter of knee, width of ankle, and the length of lower limbs were taken. According to the Davis Protocol, 22 passive reflective markers were attached to every subject’s body. All anthropometric measures and attached markers were taken by the same previously trained person. The BTS System was calibrated before the measurement, which enabled a spatial assessment of distance between the markers. The gait assessment consisted of 10 s of static capture and six walking trials (to increase reliability) on the 8 m track. Walking with the participant’s spontaneous velocity took place without any stimulation during movement; only a starting command was used. Collected static and dynamic data was utilized to generate a report containing the temporal and spatial gait parameters.

### Stability Assessment

In Romberg’s test which assessed stability, Computerized Dynamic Posturography CQStab was applied (Świerc, 2009). The recording was made twice in the static position, each of the records lasted 30 s, carried out at first with eyes opened and secondly eyes closed (Błaszczyk and Czerwisz, 2005; Ocetkiewicz et al., 2006; Strzecha et al., 2008).

### Neurologic Music Therapy Procedure

The therapeutic program for the experimental group included 4 weeks of individual 45-min sessions of NMT four times a week. At the time of the sessions the subjects were in an “on” phase (the best possible mobility). Participation in the sessions did not require any previous knowledge or musical skills. Each of the therapy sessions took place according to the same scheme. It comprised practicing activities of daily living, balance, pre-gait and gait training by using sensorimotor NMT techniques: TIMP, PSE, and RAS. For planning the therapy sessions the TDM was employed. Percussion instruments (cajon, conga, drums, maracas, and tambourine), a metronome and recorded rhythmic music were the basis for each session. Different sizes, shapes and sounds of the instruments provided numerous possibilities for motor activity stimulation.

Applied music, through its main elements – pitch, dynamics and harmony, meter, tempo and rhythm, supported the organization of movement in time and space, introduced movement fluency, gave an impulse to the muscle and provided rhythmic instructions to initiate and continue the activity. Rhythmic music, mostly African and Indian, was selected by the music therapist. The rhythmic structure of the music gave a temporal cue for movement independently of the participants’ music preferences (pure sensorimotor stimulation). For this reason participants were not asked about their music preferences. MP3 recordings were played during the session, and the volume was adjusted individually to the auditory perception of the participant. The metronome provided an additional analog auditory cue to feed the exact tempo and rhythm during pre-gait and gait excercises. In order to enhance the effect, the metronome tone was embedded into the music (Thaut, 1999; Mertel, 2014; Thaut and Rice, 2014).

### Research Protocol (NMT Training)

Warm-up exercises provided the initial part of the session. The aim was to increase the range of motion of the trunk and limbs, muscle tension adjustment (reduction of stiffness) and preparation of the whole body for further activities. Participants performed rotations of the upper and lower body, stretching exercises, and movements of the upper and lower limbs with TIMP.

Exercises of the activities of daily living (ADL) were addressed the next part of the session. Movements similar to those performed in daily life were simulated through PSE and TIMP, including turning, changing of position (moving from lying to sitting, from sitting to standing, from standing to sitting), reaching for an object to the front, above the head, reaching back, stepping up and down.

Following ADL exercises, the session focused on pre-gait training. In this section TIMP and PSE facilitated gait phases (stand or swing), step length, shifting body weight to the side and forward (balance reactions), coordination and reciprocal movements of the upper and lower limbs.

Stimulation of gait pattern provided the final part of the session. RAS was used for improving gait speed, step length, walking up and down the stairs with assistance of metronome and music. Advanced walking exercises were also practiced: initiating and stopping to the musical cues, turning, jumping, braiding, and backward gait. The session concluded with breathing exercises accompanied by relaxing and calming music (examples of NMT training – **Supplementary Figures S1**–**S3**).

### Statistical Analysis

Power calculation by Cohen (1992) methodology was utilized for this pilot study. Shapiro–Wilk test was applied to assess the normality of distribution. Since data were abnormally distributed in both groups, non-parametric tests were employed for all comparisons. In order to assess the difference in gait and stability parameters between trial I and II, a Wilcoxon matched-pairs test was applied. A Mann–Whitney *U* test was utilized to evaluate the level of significance in differentiation between the examined groups. Statistical analysis was performed using Statistica 10 Statsoft and the statistical package R 3.1.2 (R Development Core Team, 2009).

## Results

Prior to the research the distribution of characteristics within both groups selected for the study was compared. All *p*-values were higher than 0.05; thus, no significant differences were demonstrated between the two groups with regard to gender, age, duration of disease, H&Y stage scale and location of the weaker side (**Table 1**).

After the first examination of the groups, differences in terms of obtained gait and stability parameters were analyzed, presenting no significant differences between measured parameters. The summary of the results attained from the measures (performed twice in each group) is displayed in the tables and figures below. To address the research question, the outcomes of the first and second tests were compared. Then the significance of differences in obtained parameters between the groups were examined.

### Gait

In order to examine the effect of applied NMT for motor performance, changes in all the measured spatial and temporal gait parameters were thoroughly analyzed. The comparison of results of temporal and spatial parameters from trial I and II are presented in the **Tables 2** and **3**).

**Table 2:** Temporal parameters (I and II trials).

Measure	Trial	Mean	*SD*	Median	Min	Max	Q1	Q2	*p*
**(A) Experimental group**									
Stance phase (%)	I	63,53	2,63	63,1	58,3	69,4	61,4	65,03	*p* < 0,001
	II	62,19	1,5	61,9	59,5	67,1	61,13	63,2	
Swing phase (%)	I	36,37	2,79	36,9	29,3	41,7	34,82	38,6	*p* < 0,001
	II	37,81	1,5	38,1	32,9	40,5	36,8	38,88	
Double support (%)	I	13,66	2,65	12,95	9,1	20,9	11,78	15,43	*p* < 0,001
	II	12,26	1,54	11,85	9,2	16,6	11,17	13,2	
Stride time (s)	I	1,1	0,14	1,1	0,86	1,57	1	1,15	*p* < 0,001
	II	1,04	0,08	1,04	0,9	1,17	0,96	1,09	
Cadence (step/min)	I	110,38	12,65	109,8	78,1	139,8	103,3	119,32	*p* = 0,001
	II	116,64	9,13	114,7	103,3	133,9	110,1	124,58	
**(B) Control group**									
Stance phase (%)	I	63,44	1,89	63,25	58,9	70,4	62,3	64,38	*p* = 0,131
	II	63,18	1,99	63,25	59,4	68,9	61,7	64,27	
Swing phase (%)	I	36,56	1,89	36,75	29,6	41,1	35,62	37,7	*p* = 0,056
	II	36,88	1,95	36,9	31,1	40,6	35,82	38,3	
Double support (%)	I	13,56	1,86	13,3	9,6	20	12,5	14,2	*p* = 0,065
	II	13,17	2,05	13,05	8,9	18,1	11,77	14,07	
Stride time (s)	I	1,08	0,14	1,08	0,84	1,52	1,01	1,16	*p* = 0,438
	II	1,08	0,12	1,08	0,86	1,32	1	1,16	
Cadence (step/min)	I	112,5	14,07	111,6	79,95	142,1	103,9	119,9	*p* = 0,545
	II	113,06	12,78	111	91,4	138,7	103,6	119,7	


**Table 3:** Spatial parameters (I and II trials).

Measure	Trial	Mean	*SD*	Median	Min	Max	Q1	Q2	*p*
**(A) Experimental group**									
Step lenght (m)	I	0,48	0,11	0,5	0,19	0,7	0,42	0,54	*p* < 0,001
	II	0,55	0,1	0,55	0,24	0,75	0,5	0,6	
Velocity (m/s)	I	0,99	0,27	1,01	0,4	1,51	0,83	1,18	*p* < 0,001
	II	1,2	0,22	1,23	0,52	1,62	1,1	1,33	
Stride length (m)	I	1,07	0,24	1,12	0,46	1,48	0,94	1,22	*p* < 0,001
	II	1,23	0,2	1,24	0,6	1,56	1,18	1,35	
Step width (m)	I	0,15	0,02	0,14	0,11	0,19	0,14	0,16	*p* = 0,172
	II	0,15	0,02	0,15	0,11	0,2	0,13	0,16	
**(B) Control group**									
Step lenght (m)	I	0,5	0,05	0,5	0,34	0,6	0,48	0,54	*p* = 0,035
	II	0,52	0,07	0,54	0,35	0,62	0,46	0,57	
Velocity (m/s)	I	1,04	0,19	1,06	0,69	1,37	0,89	1,17	*p* = 0,03
	II	1,08	0,21	1,1	0,66	1,49	0,98	1,2	
Stride length (m)	I	1,1	0,12	1,12	0,81	1,32	1,07	1,19	*p* = 0,038
	II	1,14	0,15	1,17	0,79	1,37	1,05	1,25	
Step width (m)	I	0,15	0,02	0,16	0,11	0,2	0,14	0,17	*p* = 0,107
	II	0,15	0,03	0,15	0,02	0,2	0,14	0,17	


### Analysis of Temporal Gait Parameters

The second measure of the temporal gait parameters was significantly higher than the first for the duration of swing phase and cadence. The second measure of duration of stance phase, the double support time and the stride time was significantly lower than the first. The comparison of the results of temporal gait parameters in the control group did not reveal any statistically significant differences between the trial I and II (**Table 2**).

### Analysis of Spatial Gait Parameters

The second measure of spatial gait parameters was significantly higher than the first one for the step length, velocity and the stride length. Analysis of the results for the same parameters in the control group also demonstrated significant differences, but the significance level was lower than in the experimental group. Step width did not differ significantly in any of the examined groups (**Table 3**).

### Comparison of Changes in Temporal Parameters Between the Groups

**FIGURE 1:**
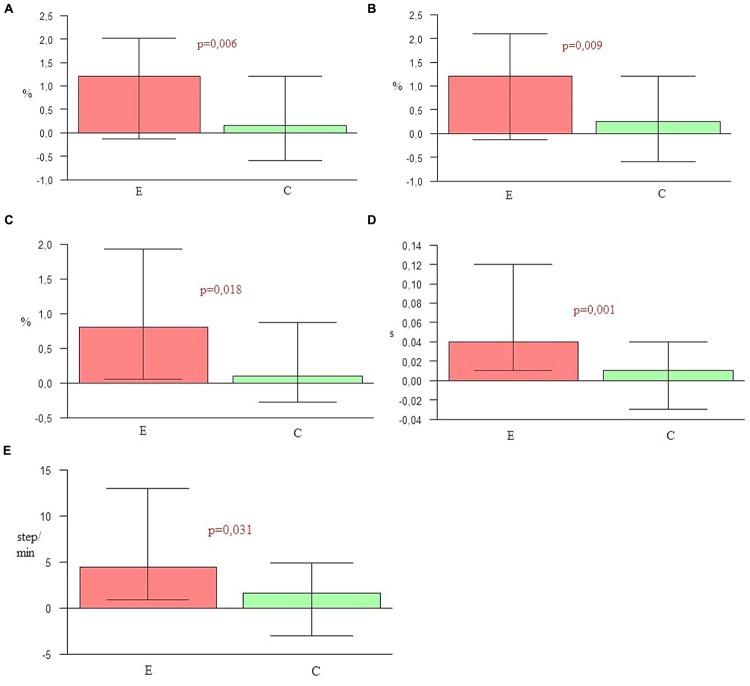
**(A)** Shortening of stance phase. **(B)** Extension of swing phase. **(C)** Shortening of double support. **(D)** Shortening of stride time. **(E)** Increasing of cadence.

In the experimental group the shortening of the parameters such as stance phase, double support time, and stride time was more considerable than in the control group. Moreover, for the subjects from the experimental group the extension of the swing phase and increasing of cadence was significantly higher than for the controls (**Table 4** and **Figure 1**).

**Table 4:** Temporal parameters – groups comparison (E and C).

Changes of measure	Group	Mean	*SD*	Median	Min	Max	Q1	Q2	*p*
Shortening of stance phase (%)	E	1,34	2,08	1,2	–2,4	7,2	–0,12	2,02	*p* = 0,006
	C	0,26	1,64	0,15	–6,6	5,3	–0,6	1,2	
Extension of swing phase (%)	E	1,45	2,25	1,2	–2,4	7,9	–0,12	2,1	*p* = 0,009
	C	0,32	1,62	0,25	–6,6	5,3	–0,6	1,2	
Shortening of double support (%)	E	1,4	2,23	0,8	–2	8,8	0,05	1,93	*p* = 0,018
	C	0,39	1,43	0,1	–4,8	3,8	–0,27	0,88	
Shortening of stride time (s)	E	0,07	0,1	0,04	–0,07	0,42	0,01	0,12	*p* = 0,001
	C	0,01	0,07	0,01	–0,12	0,21	–0,03	0,04	
Increasing of cadence (step/min)	E	6,26	8,4	4,45	–6,8	26,2	0,85	12,95	*p* = 0,031
	C	0,56	6,72	1,6	–13,9	11,45	–3	4,9	


### Comparison of Changes in Spatial Parameters Between the Groups

**FIGURE 2:**
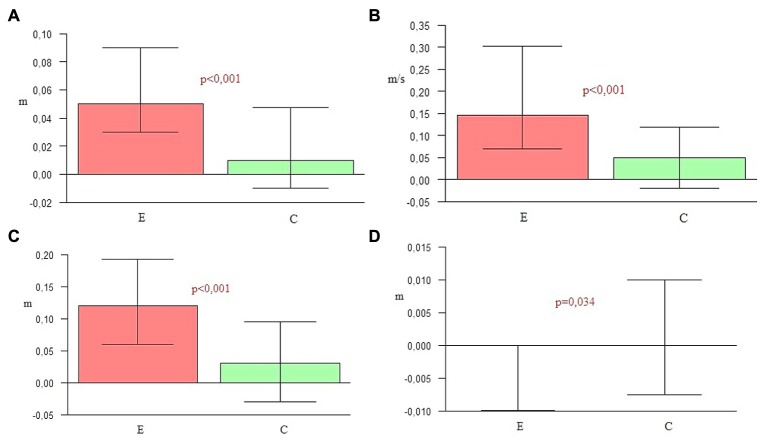
**(A)** Elongation of step length. **(B)** Increasing of velocity. **(C)** Elongation of stride length. **(D)** Increasing of step width.

A significance level lower than 0.001 indicated a statistically significant difference in the elongation of both step and stride length in the experimental group in comparison to the control group. An increase of velocity in the experimental group was also higher than in the control group. Furthermore, a significant difference in the increase of step width observed in the control group was higher than for the experimentals (**Table 5** and **Figure 2**).

**Table 5:** Spatial parameters – groups comparison (E and C).

Changes of measure	Group	Mean	*SD*	Median	Min	Max	Q1	Q2	*p*
Elongation of step length (m)	E	0,06	0,09	0,05	–0,16	0,38	0,03	0,09	*p* < 0,001
	C	0,02	0,05	0,01	–0,1	0,13	–0,01	0,05	
Increasing of velocity (m/s)	E	0,21	0,2	0,14	–0,24	0,75	0,07	0,3	*p* < 0,001
	C	0,04	0,13	0,05	–0,25	0,31	–0,02	0,12	
Elongation of stride length (m)	E	0,16	0,18	0,12	–0,21	0,83	0,06	0,19	*p* < 0,001
	C	0,03	0,11	0,03	–0,22	0,26	–0,03	0,1	
Increasing of step width (m)	E	0	0,01	0	–0,03	0,02	–0,01	0	*p* = 0,034
	C	0,01	0,03	0	–0,02	0,17	–0,01	0,01	


**Figures 1** and **2** graphically depict significant differences in the changes of gait parameters between the groups, demonstrating the efficacy of the applied therapy. The second gait measure in the experimental group was significantly different for the majority of parameters in comparison to the first one. There were also statistically significant differences in the comparison of both groups. These outcomes indicate the effectiveness of the research protocol employed to improve the temporal and spatial parameters of gait in PD.

### Stability

In order to determine the effect of NMT on stability, the changes in the parameters obtained in the Romberg’s test (with eyes open and eyes closed) were examined. As in the case of the gait parameters, a comparison of the results of the trial I and II, the experimental and the control group was conducted. Also the differences between the groups were analyzed.

### Stability Parameters from the Test with Eyes Opened

**FIGURE 3:**
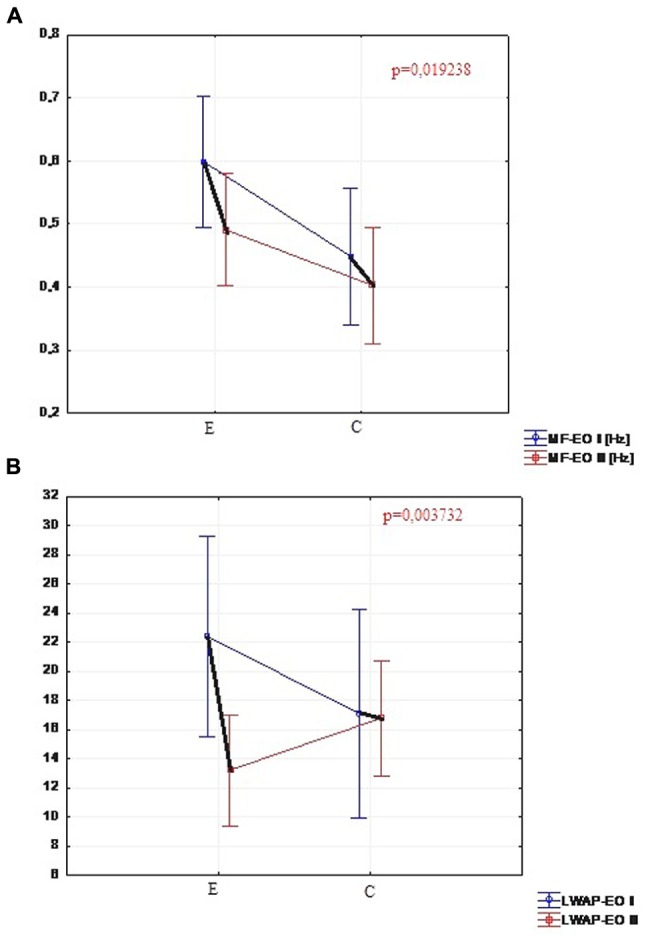
**(A)** Comparison of MF-EO in the experimental and control group (I and II trials). **(B)** Comparison of LWAP-EO in the experimental and control group (I and II trials).

Out of 17 measured parameters in the test with eyes open, only two changed significantly in the experimental group. The change concerned the center of pressure mean frequency (MF-EO) measured in Hz (the level of significance *p* = 0.019) and the amount of sway in sagittal plane (LWAP-EO) by center of pressure (the level of significance equal to 0.003). There was no significant difference demonstrated in the comparison of the results of the same parameters in the control group. In the control group only one of the parameters changed significantly (**Figure 3**). It was the mean velocity (MVML-EO) of the center of pressure in frontal plane on the level of significance *p* = 0.049.

### Stability Parameters from the Test with Eyes Closed

**FIGURE 4:**
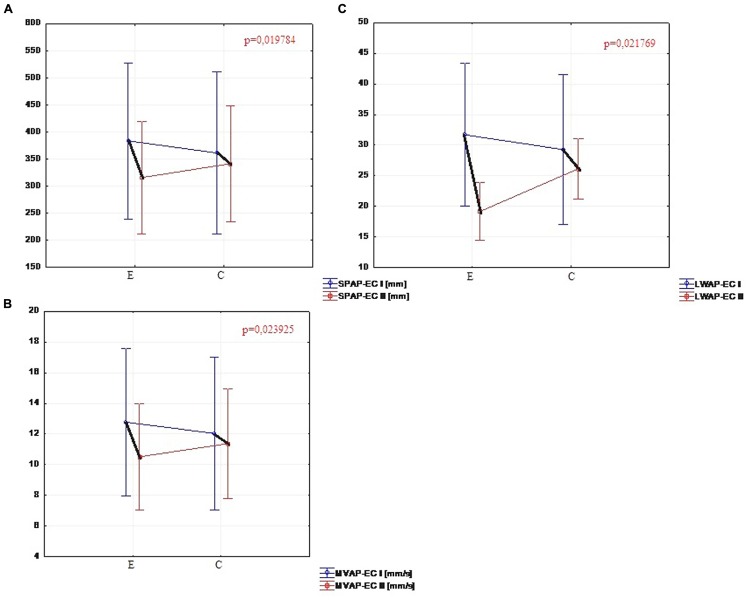
**(A)** Comparison of SPAP-EC in the experimental and control group (I and II trials). **(B)** Comparison of MVAP-EC in the experimental and control group (I and II trials). **(C)** Comparison of LWAP-EC in the experimental and control group (I and II trials).

The comparison of the test results with eyes closed in the experimental group showed significant changes in five parameters. In the second trial the total sway path (SP-EC) calculated in both planes (*p* = 0.032), the sway path (SPAP-EC) in milimeters calculated in sagittal plane (*p* = 0.019), the mean velocity of the center of pressure (MV-EC) in both planes (*p* = 0.035), the mean velocity of the center of pressure (MVAP-EC) in the sagittal plane (*p* = 0.023) and the amount of sway (LWAP-EC) in sagittal plane (*p* = 0.021) were significantly reduced (**Figure 4**). The comparison of the results of the same parameters for the control group showed no significant difference, while statistically significant differences were observed in the sway path (SPML-EC) in frontal plane (*p* = 0.031) and the mean velocity of the center pressure (MVML-EC) in frontal plane (*p* = 0.025).

### Group Comparisons

The comparison of changes in parameters of Romberg’s test between the two groups displayed no significant differences either in the test with eyes open or with eyes closed. The only parameter that was significantly reduced in the experimental group (compared to the controls) was the amount of sway (LWAP-EO) in sagittal plane in a test with eyes opened.

The results obtained from Romberg’s test with eyes opened changed significantly in the experimental group only with regard to two parameters. Due to these outcomes it is impossible to confirm the effectiveness of the research protocol in the improvement of the stability of people with PD.

Significant changes in the five parameters with eyes closed in the sagittal plane might indicate an improvement of proprioception (a part of somatic sensory system responsible for the sense of body position and movement – Purves et al., 2004) and body perception in the same group of patients.

## Discussion

The key hypothesis of this project assumes the major role of the combination of all three NMT sensorimotor techniques in improving spatio-temporal gait parameters and postural stability in the course of PD. During 4 weeks of a rehabilitation program based on sensorimotor NMT techniques conducted with PD patients, functional movements, stability and locomotion were stimulated with methods incorporating the use of music with a strong sense of rhythm. To confirm the first part of the hypothesis, nine spatio-temporal parameters of gait were calculated and compared by the Optoelectrical 3D Movement Analysis System BTS Smart. The comparison of these results in the experimental group before and after NMT sessions showed statistically significant changes in the majority of parameters (*p* < 0.05). The second measure demonstrated that the duration of the swing phase, cadence, step length, velocity and stride length were significantly higher than in the first one. In the second measure the duration of the stance phase, the time of double support and the stride time were significantly lower than in the first one. Only the width of the step did not change significantly, but this parameter depends mostly on individual anatomy. The second measure of the gait parameters in the control group was significantly higher than the first one only for three parameters: the step length, velocity, and stride length. The comparison of the outcome measures between the two groups showed that in every parameter *p*-values were lower than 0.05. In the experimental group a significant shortening of stance phase (*p* = 0.006), elongation of swing phase (*p* = 0.009), shortening of the time of double support (*p* = 0.018), shortening of stride time (*p* = 0.001), the increase of cadence (*p* = 0.031), elongation of step length (*p* < 0.001), the increase of velocity (*p* < 0.001), and elongation of stride length (*p* < 0.001) was observed in comparison to the control group. In the control group only the step width (*p* = 0.034) was higher than in the experimental group. This may be a sign of increasing balance disorders in the control group, resulting in the widening of the base of suport. The obtained outcomes confirm the high efficiency of the applied NMT. The sensorimotor techniques employed in this study significantly differentiated the experimental group and the control group in terms of the measured gait parameters.

Currently, the foundation of gait treatment in PD is the application of pharmacology, supported by training with cueing. Research shows that reduced joint amplitude caused by the illness can be normalized through receiving levodopa and cues, which in turn results in the improvement of motor behavior and a bypassing of the damaged motor mechanisms in the basal ganglia (Morris et al., 2005). In this study, rhythmic auditory cues were applied in the experiment to improve gait. It should be noted that it is very important to choose an appropriate rhythmic stimulation. Optimal rhythmic stimulation, adjusted to the patient’s preferred velocity, ranges from 60 to 150 beats per minute. A metronome tempo set below each clients preferred velocity reduces stability, whilst a pace above this range negatively affects step length (Ebersbach et al., 1999; Arias and Cudeiro, 2008). Similarly, rhythmic cueing is most effective when music is selected for bringing clear rhythmic instructions that controls the pace and cadence of gait (Brown et al., 2009). The music of patient’s preference is not always suitable for RAS; therefore, the selection is usually made by a music therapist. For the purposes of this study, the selection of suitable music for physical stimulation was also conducted by the leading music therapist. The ability of human brain to entrain with the rhythm of movement allowed the subjects to react on the beat, even if the selected music did not quite match the taste of the participants.

Previously, most researchers tended to focus on only one of the NMT techniques – RAS – for gait facilitation in PD (McIntosh et al., 1997; Freedlanda et al., 2002; Hausdorff et al., 2007); whereas the authors of this study evaluated the effectiveness of NMT protocol with not only RAS, but an approach combining the two remaining sensorimotor NMT techniques: TIMP and PSE. The authors decided not to measure the gait parameters during rhythmic stimulation which would have revealed the immediate effect of the rhythmic auditory cues. Instead, they examined the effect of musical and rhythmic stimulation after a 4-week therapy cycle. Consequently, during the first and second measure patients were moving independently, without any cues during the movement; they received only the starting command. The second measure was taken the day after the therapy was concluded. According to available publications, the effect of the RAS is maintained for several weeks after the completion of this form of therapy. This fact was noted, among others, in the Benoit et al. (2014) study. The measure of gait performed a month after the completion of the applied rhythmic therapy, still demonstrated statistically significant changes in the velocity and length of steps. McIntosh stated that the application of RAS temporarily improves gait parameters. The patient is still able to follow the rhythm, even though the RAS disappears. This effect persists for up to 6 weeks (McIntosh et al., 1997; Thaut et al., 1998).

In addition to the forward gait analysis, it is worth examining turning during gait. Due to the close relationship between gait and balance disorders, turning often presents considerable difficulties for PD patients. Unfortunately, the pathophysiology of these difficulties is still poorly understood (Dibble et al., 2008). In his study, Huxham et al. (2008) dealt with analysis problems with turning during walking. 20 patients (10 with PD and 10 healthy subjects of similar age) were examined with the use of 3D motion analysis system. The study focused on the spatiotemporal regulation of steps while turning up to 60–120°. Significant differences in most factors were noted in the PD group. The spatial regulation of turning was similar, even if slightly reduced, however, the velocity of turning was slower than in the control group. In general steps are shorter during walking in PD, but there was a significant additional reduction during turning; there were small but significant differences in the regulation of the steps in time. The differences between the groups, more visible in the performance of larger turns, may reflect balance and coordination impairment in people with PD during demanding gait tasks (Woollacott and Shumway-Cook, 2002). These findings prompted the authors of this study to formulate the research question concerning the changes of stability under the influence of applied NMT. According to another study (Koh et al., 2008), the reasons for main changes within velocity and step length can be related to problems with postural stability that appear with the development of the disease. The gait assessment in PD patients with postural instability showed a significant reduction in velocity and step length in comparison to a group with tremor. Also pelvic and lower limb joints range of motion were significantly reduced in this group. Reduced pelvic motion range may be a compensatory strategy for postural instability which provokes shortening of step length in PD patients, which in turn affects the velocity of gait.

The second part of this study – the influence of research protocol on stability, cannot be verified positively. There were no significant differences between the first and the second posturography measure within the experimental group, or between the groups. This suggests that the application of NMT primarily leads to an improvement of rhythmical movements, with center of mass transfered forward or backward (walking, daily activities). The static stability tested with eyes open did not change significantly for the majority of measured parameters. Significant differences in the experimental group were reported only for: the center of pressure mean frequency (MF-EO) measured in Hz and the amount of sway in the sagittal plane (LWAP-EO). Therefore, this type of training may not be so effective for improving stability parameters with visual control. However, during the research cycle the stability did not deteriorate. This type of therapy may help to maintain the satisfactory level of stability, thereby delaying balance disorder that often occurs at the beginning of the third stage of PD in the H&Y scale.

What was particularly interesting in the study, was the occurance of the statistically significant differences (*p* < 0.05) in the stability tests with eyes closed in the experimental group within five parameters: the total sway path (SP-EC) calculated in both planes, the sway path (SPAP-EC) in milimeters calculated in sagittal plane, the mean velocity of the center of pressure (MV-EC) in both planes, the mean velocity of the center of pressure (MVAP-EC) in the sagital plane and the amount of sway (LWAP-EC) in sagital plane. The transfer of body weight during performance of many everyday activities, including walking, is organized also in the sagittal plane. The improvement of stability with the exclusion of a strong visual component can be the sign of proprioception and body perception enhancement through auditory stimulation, necessary to maintain balance, posture, and motor control mechanisms.

Drawing on the Koh et al.’s 2008 investigation of the impact of poor postural control on stride length and velocity, it can be expected that the improvement of these spatial parameters of gait positively affects postural control processes based on proprioceptive information. It is possible that the application of auditory stimulation exclusively is not sufficient for stability and balance training in PD.

Stożek et al. (2003) in their study evaluated the rehabilitation program focused on improving balance in PD. They applied the combination of cues including verbal, visual, auditory, and proprioceptive stimulation. Posturography was employed to assess stability and balance. The stability tests were conducted with eyes open and eyes closed conditions, and the body weight transfer was measured in six directions. As with the results obtained by the authors of this article, the comparison of the outcomes from three different measures showed statistically significant differences in the stability parameters only with eyes closed. These differences were observed between the first, the second, and the third measure. The last measure was carried out a month after the completion of the training. The changes remained significant which indicated that the treatment effects were maintained. Therefore, the development of the training with closed eyes in the context of improving stability and balance in PD appears to be worthy of further exploration.

The latest scientific research carried out in the animal model of PD (Farley et al., 2008) illustrated that physical exercises with different stimuli are as efficient as a physiotherapy method, modifying the course of the disease and contributing to the functional improvement of patients. Furthermore, researchers place emphasis on the significant influence of music and rhythm on motor function, gait, mobility, and patient’s quality of life (Paccetti et al., 2000; Howe et al., 2003; Moore et al., 2006; Leins et al., 2009; De Bruin et al., 2010; Pasek et al., 2010). Therefore, the extensive application of sound and rhythmic stimulation in the basic rehabilitation program for patients struggling with the PD is supported by these pilot level findings.

This pilot level study has some limitations which merit attention. In particular, it is impossible to distinguish the individual effects of the applied techniques and indicate the most efficient for gait and balance training. Given the effectiveness of RAS has been established (McIntosh et al., 1997; Thaut et al., 1998; Hausdorff et al., 2007), future research focused on the comparative effectiveness of PSE and TIMP individually and in combination is indicated, to understand more about the characteristic effects and mechanisms of each treatment and how they might work together. Furthermore, as this data only provides an indication of short term effects, further studies are indicated for determining what long-term NMT treatment effects might be afforded through a more longitudinal design. The hypothesis assumed only efficacy of NMT in PD treatment, but did not indicate it as a superior method. The second step of the research could perhaps detect the differences between effectiveness of NMT and another chosen approach.

## Conclusion

This pilot study indicates that NMT sensorimotor techniques may be employed to improve gait and other rhythmical activities for individuals with PD. Changes in stability without visual control indicate improvement of proprioception, giving a new compensatory strategy for movement and postural control through auditory system. The confirmation of the research hypothesis in this study builds the evidence base for a therapeutic strategy based on the use of rhythmic music for the improvement and maintenance of good functional state. These changes may be effective in improving patient’s ability to perform activities of daily living and engage in social activities important for their quality of life.

This study illustrates that by connecting the disciplines of music therapy, physiotherapy and occupational therapy, through the work of task orientation, including transfers, activities of daily living and locomotion, as well as providing an appropriate level of difficulty and number of repetitions, it may be possibile to decrease damaging effects of PD. However, the question of how to prolong the obtained effect of rhythmic and musical stimulation requires further investigation.

## Conflict of Interest Statement

The authors declare that the research was conducted in the absence of any commercial or financial relationships that could be construed as a potential conflict of interest.

## Supplementary Material

**FIGURE S1 — Balance exercise - reaching back with the trunk rotation.**

**FIGURE S2 — Gait preparation - reciprocal movement of upper and lower extremities.**

**FIGURE S3 — Gait preparation - steps forward-backward with arms swing.**
