# Supplementary figures and images for: Neurologic Music Therapy Training for Mobility and Stability Rehabilitation with Parkinson’s Disease – A Pilot Study
Source: Front Hum Neurosci. 2016 Jan 26;9:710. doi: 10.3389/fnhum.2015.00710 (PMC4726780; doi:10.3389/fnhum.2015.00710)

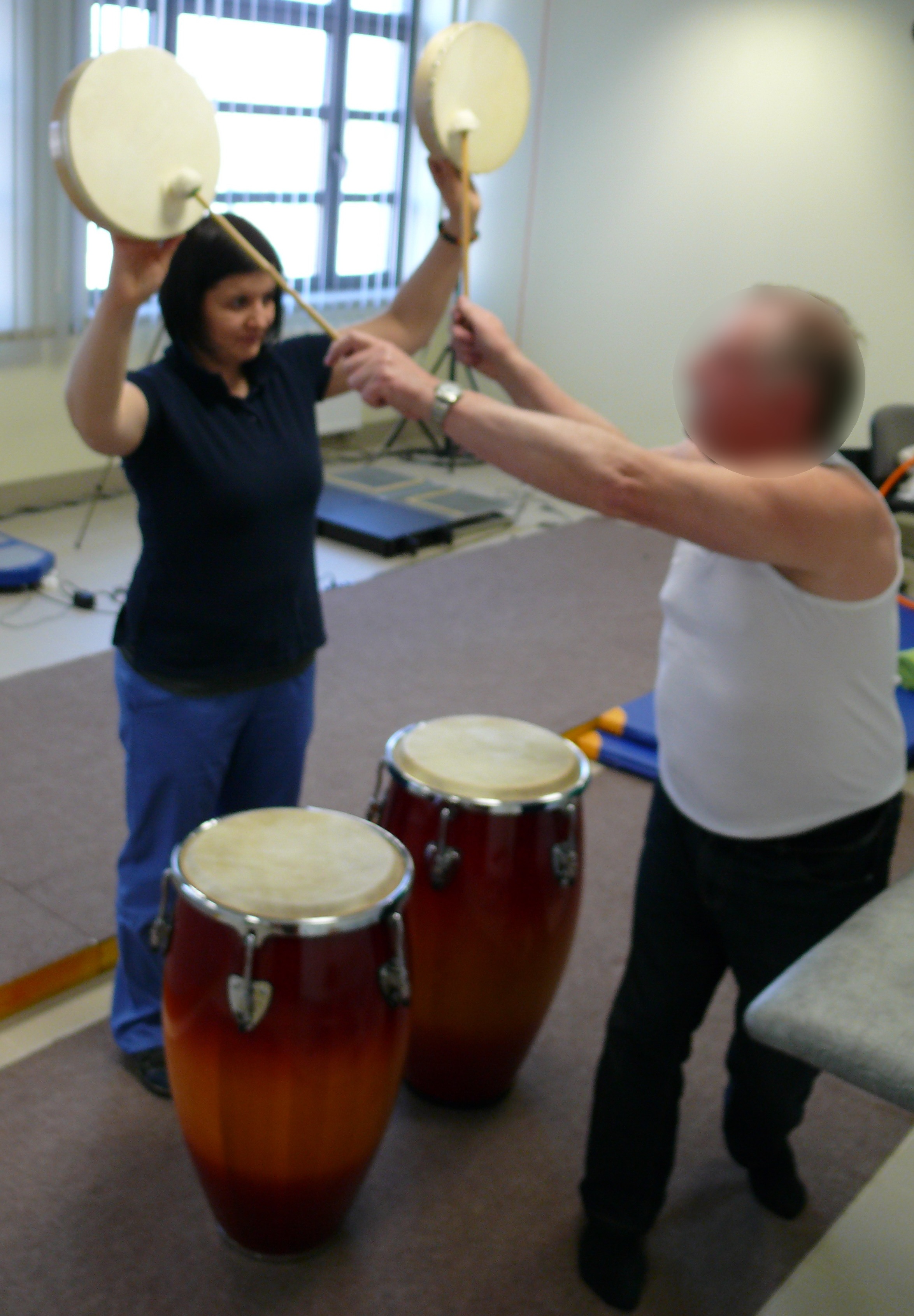

Supplement: Supplementary file 1 [file Image_1.JPEG]

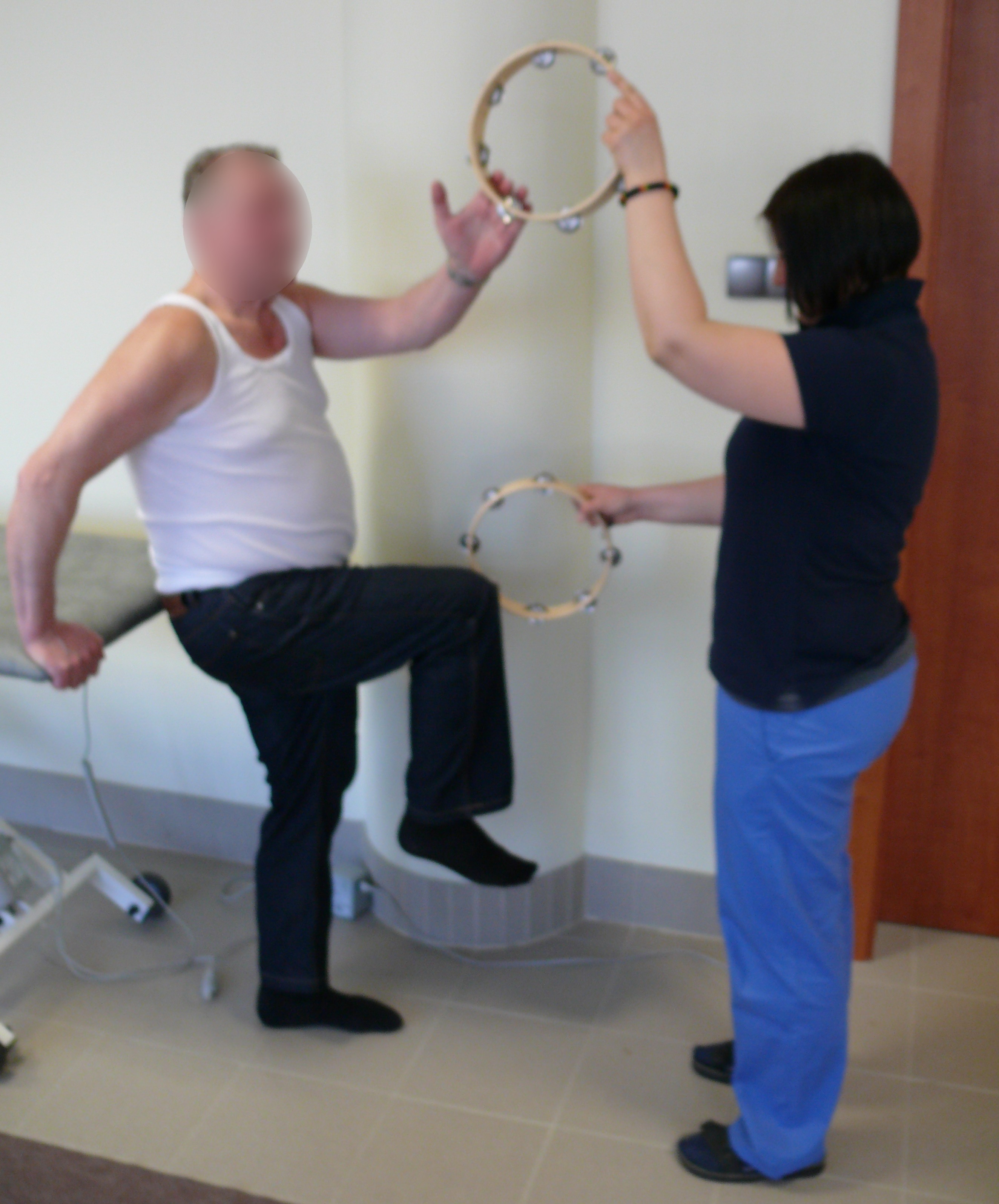

Supplement: Supplementary file 2 [file Image_2.JPEG]

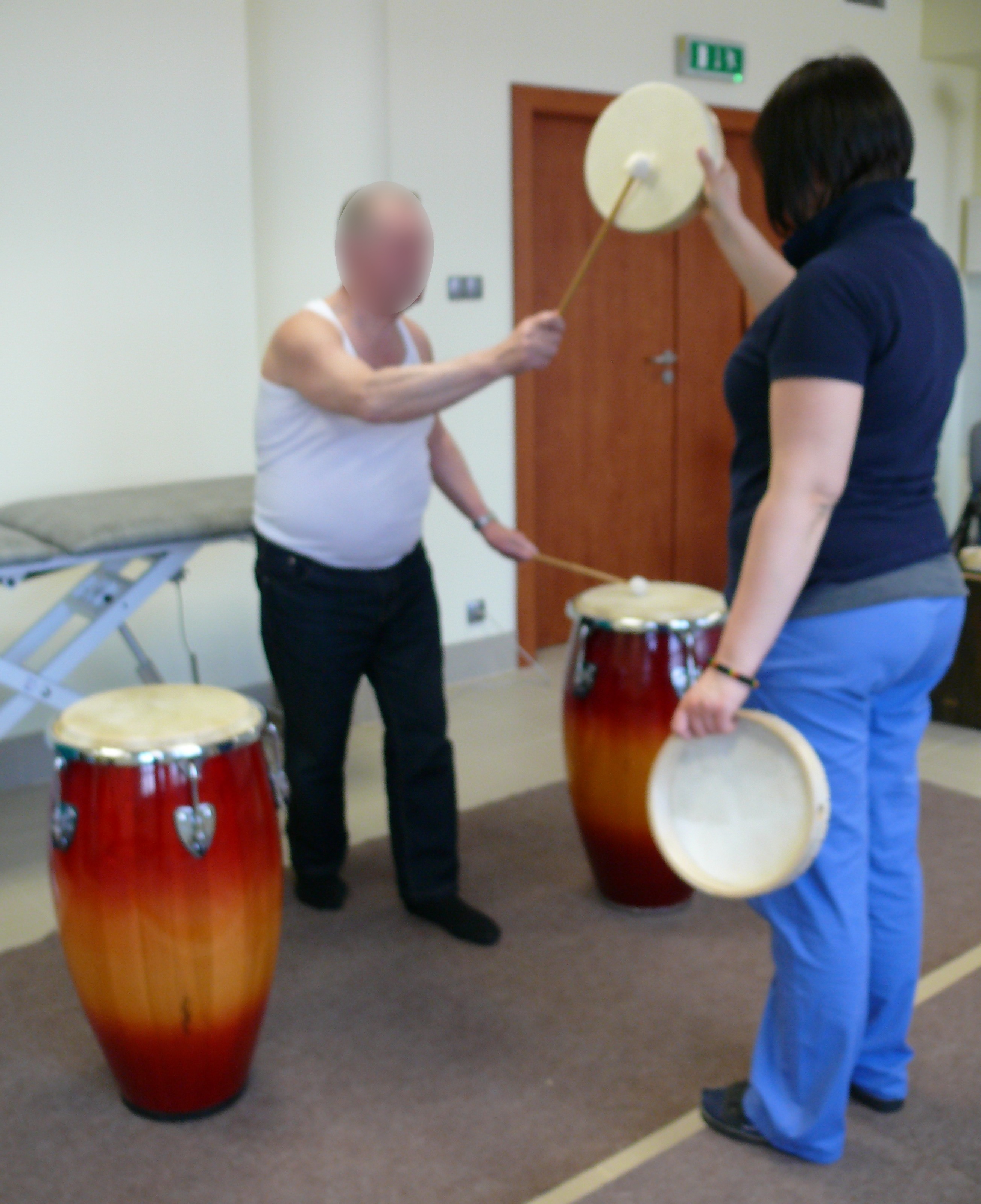

Supplement: Supplementary file 3 [file Image_3.JPEG]
